# Supplementary material for: A double-blind replication attempt of offline 5Hz-rTUS-induced corticospinal excitability
Source: Imaging Neurosci (Camb). 2025 Dec 10;3:IMAG.a.1046. doi: 10.1162/IMAG.a.1046 (PMC12696668; doi:10.1162/IMAG.a.1046)
Supplement: Supplementary Material [file IMAG.a.1046_supp.pdf]

Supplementary Material

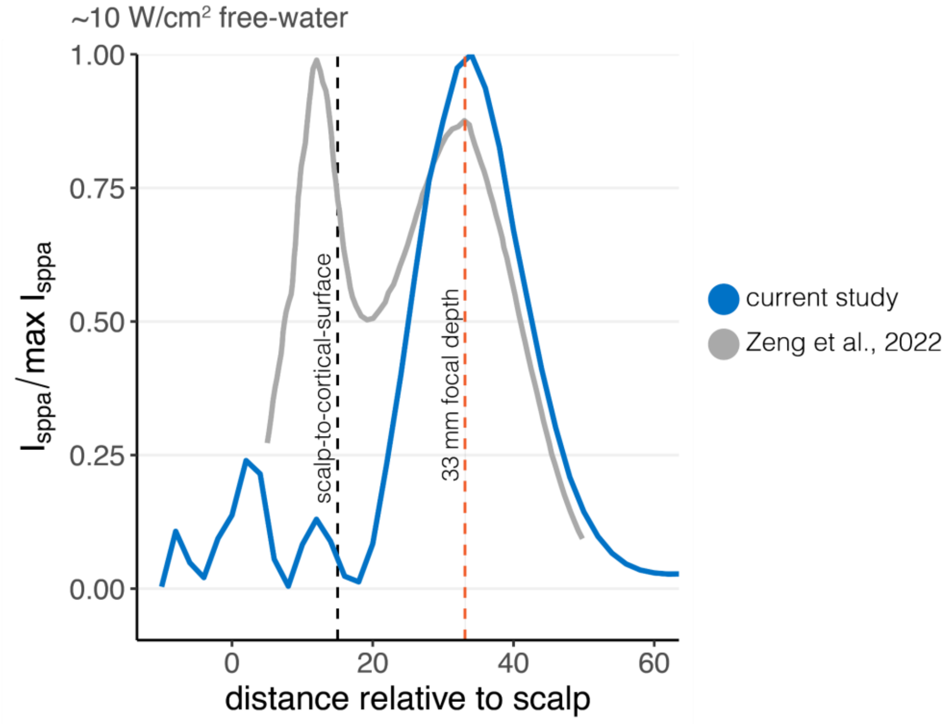

Supplementary Fig. 1. Comparison of normalised axial intensity profiles between present and prior work. The peak intensity of the intended focus using transducer H246 was 33 mm for Zeng and colleagues (2022; dashed orange line). We replicate the intended focal depth of Zeng et al (2022) by using a TPO focal depth setting of 43.5 mm for CTX500-025, and accounting for the use of a 10 mm gel pad. We show a similar axial profile. An average scalp-to-cortical-surface distance of 15 mm is depicted with a dashed black line(Osada et al., 2022). Plots adapted from the original report(Zeng et al., 2022).

For the present study, the axial profiles for CTX500-025 measured by the manufacturer (Sonic Concepts, WA, USA) at similar focal depths (grey) were interpolated to estimate the pressure distribution for the currently applied focal depth of 43.5 mm (blue). The normalised axial profile data displayed in the original study (Zeng et al., 2022) were extracted using WebPlotDigitizer (version 5; CA, USA) by digitising the axes, performing automatic extraction using a pen tool and a colour filter, and sampling at a 5-pixel interval.

Supplementary Table 2

Transducer and Drive System Parameters

	Manufacturer, Model Number	Centre Frequency	Radius of curvature	Aperture Diameter	Number of Elements	Element Distribution
Transducer	Sonic Concepts, CTX-500-025	500 kHz	64 mm	64 mm	4	Spherical cap, annular array, equal area,
Matching	4 channel electrical impedance matching network, Sonic Concepts					
Drive system	NeuroFUS Pro 4 channel TPO					

Drive System Settings

	Operating Frequency	Output level Setting	Focal Position Setting
Motor	500 kHz	10 W/cm^2	43.5 mm

Free Field Pressure Parameters

	Spatial Peak Pressure Amplitude	Position of Spatial Peak Pressure	Axial Focal Size (-3dB)	Lateral Focal Size (-3dB)	Axial Focal Size (-6dB)
43.5mm	548 ± kPa	[0, 0, 53.5] mm	14.2 mm	2.6 mm	19.7 mm

Notes: Reference position is at centre of the spherical array surface.

Pulse Timing Parameters

		Duration	Ramp Duration	Ramp Shape	Repetition Interval / Frequency
Experiment 2	Pulse	20 ms	0 ms	No ramp	0.2 s / 5 Hz
	Pulse Train	80 s			

Notes:

In Situ Exposure Parameters

	Spatial Peak Pressure Amplitude [kPa]	Spatial peak pulse average intensity [W/cm2]	Mean position of Spatial Peak Pressure [mm]	Axial Focal Size (-3dB) [mm]	Lateral Focal Size (-3dB) [mm]	Axial Focal Size (-6dB) [mm]
Motor	189 ± 39	1.2 ± 0.43	[0.3 ± 0.5, -0.1 ± 0.5, 51.1 ± 1.3] mm	17.0 ± 3.1	[3.1 ± 0.2, 2.9 ± 0.1]	32.9 ± 14.3

Notes: Reference position is at centre (origin) of spherical array surface

Mechanical Index	Maximum temperature rise
0.26 ± 0.05	0.22 °C

M1 ROI

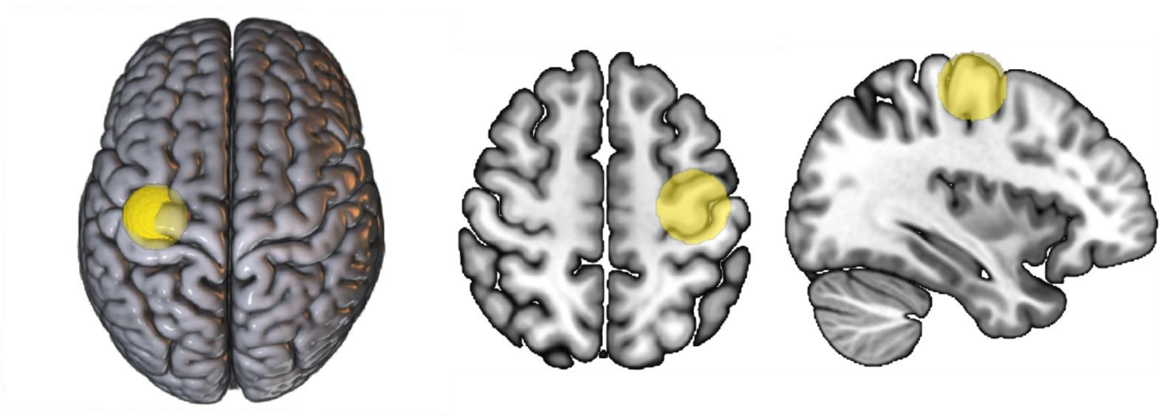

Supplementary Fig. 2. **15 mm M1 spherical region-of-interest (ROI).** The ROI was structurally identified in MNI152 space and used to assess targeting. The MNI coordinators is -34, -16, 58. The ROI was placed such that the both the lip/crown of the precentral gyrus and the omega formation are included.

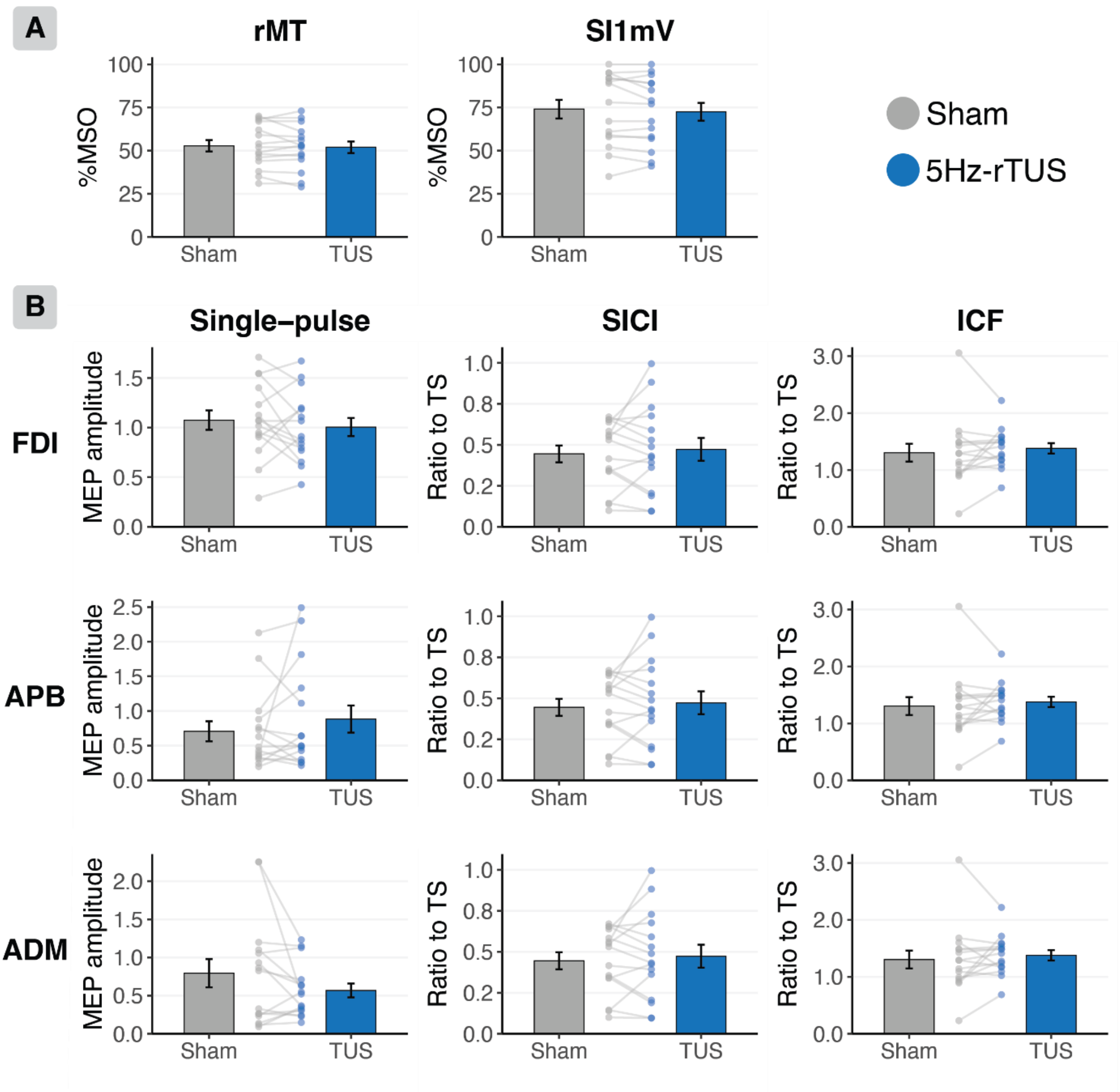

Supplementary Fig. 3. No significant differences between sham and 5Hz-rTUS at baseline

A. Sham vs 5Hz-rTUS effects on resting motor threshold (rMT) and the TMS intensity required to evoke a ~1 mV MEP (SI_{1mV}).

B. Single-pulse MEP amplitude (left), short interval cortical inhibition (SICI; middle), and intracortical facilitation (ICF; right) measured from the FDI (top), APB (middle), or ADM (bottom) muscles, respectively.

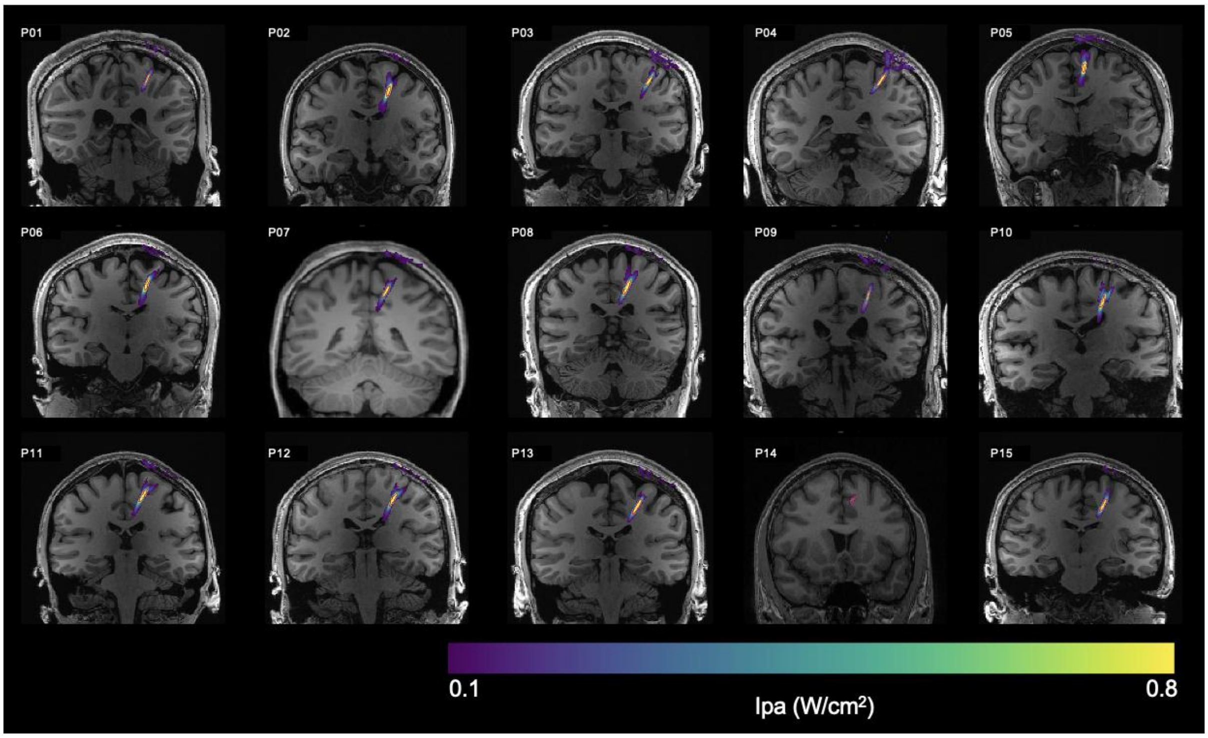

Supplementary Fig. 4. The simulated acoustic fields for individual participants. Individual simulations of acoustic wave propagation thresholded at 0.1-0.8 W/cm^2. Circled in red is the acoustic focus for each subject, defined as the full-width half maximum intensity (FWHM).

Supplementary Information 1

The hair preparation and the estimated additional thickness of 0.5mm after hair preparation.

We performed the hair preparation according to the practical guide from ITRUSST (Murphy et al., 2025), which introduced degassed ultrasound gel on the scalp to penetrate between the hair strands. This will form a thin layer of hair and gel on the scalp. However, it is impossible to measure the thickness accurately after hair preparation and placement of the transducer on the scalp. Therefore, we estimate the 0.5mm according to the 0.08~0.1mm of averaged hair diameter across African, Asian and Caucasian(Franbourg et al., 2003; Robbins, 2002) , 6-10% of the hair swelling rate in water, and 4 times of hair preparation(Murphy et al., 2025) . So, the estimated hair thickness by 4 layers of the swelled hair (0.1mm x 1.1 ratio of swelling x 4 time hair preparation = 0.44 mm after hair preparation). After considering the volume of the gel, we set 0.5mm as the hair thickness after hair preparation.

Regarding the gel pad, we employed Aquasonic gel pad (Aquaflex, Parker Laboratories, NJ, USA). It is a circular pad with 2cm thickness and 9cm of diameter. We sliced the gel pad into two pieces of 1cm in thickness and 9cm in diameter. Several previous works applied the gel pads(Yaakub et al., 2024; Yaakub et al., 2023).

Supplementary Information 2

Replicated statistical methodology (RM-ANOVAs)

Repeated measures ANOVAs (RM-ANOVA) on raw MEP amplitudes were conducted to replicate the statistical methods employed by Zeng and colleagues(Zeng et al., 2022). To examine the time course of corticospinal excitability changes in MEP amplitudes for the primary muscle of interest, the FDI, a RM-ANOVA with factors Condition (5Hz-rTUS/sham), Timepoint (Baseline/T5/T30/T60), and their interaction was conducted. This analysis revealed a trend for Timepoint ($F(1.77,23) = 2.967$, $p = 0.077$, $\eta_p^2 = 0.186$), but no significant effect of Condition ($F(1,13) = 1.903$, $p = 0.191$, $\eta_p^2 = 0.128$), nor a significant Condition*Timepoint interaction ($F(1.57,20) = 0.284$, $p = 0.703$, $\eta_p^2 = 0.021$). To further examine TUS-induced changes in corticospinal excitability, MEP amplitudes were expressed as a ratio to baseline and tested with the factors Condition (5Hz-rTUS), Timepoint (T5/T30/T60), and their interaction. No significant effects were observed (Timepoint: $F(1.33,17) = 2.465$, $p = 0.128$, $\eta_p^2 = 0.159$; Condition: $F(1,13) = 0.204$, $p = 0.659$, $\eta_p^2 = 0.015$; Timepoint*Condition: $F(2,26) = 0.045$, $p = 0.956$, $\eta_p^2 = 0.003$). In line with these findings, no significant effects were observed for paired-pulse measures (SICI: Timepoint: $F(3,42) = 0.463$, $p = 0.71$, $\eta_p^2 = 0.032$; Condition: $F(1,14) = 0.008$, $p = 0.932$, $\eta_p^2 = 0.001$; Timepoint*Condition: $F(3,42) = 0.819$, $p = 0.491$, $\eta_p^2 = 0.055$; ICF: Timepoint: $F(3,42) = 1.107$, $p = 0.357$, $\eta_p^2 = 0.073$; Condition: $F(1,14) = 2.283$, $p = 0.153$, $\eta_p^2 = 0.14$; Timepoint*Condition: $F(1.76,25) = 0.055$, $p = 0.929$, $\eta_p^2 = 0.004$). Taken together, results from closely replicated analyses do not provide evidence for effective ultrasonic neuromodulation of corticospinal excitability, in line with the linear mixed models used in the main text.

Supplementary Information 3

Results for abductor pollicis brevis (APB)

A linear mixed model predicting square root corrected MEP amplitude measured over the APB by Condition (5Hz-rTUS/sham), Timepoint (Baseline/T5/T30/T60) and their interaction revealed a significant effect of Timepoint ($F(3,14) = 4.618$, $p = 0.019$, $\eta_p^2 = 0.498$), with MEP amplitudes increasing over time. However, no significant effect of Condition ($F(1,14) = 0.123$, $p = 0.731$, $\eta_p^2 = 0.009$) or Timepoint*Condition ($F(3,14) = 1.102$, $p = 0.381$, $\eta_p^2 = 0.191$) was observed. When testing MEP amplitude expressed as a ratio to baseline, no significant effects were observed (Timepoint: $F(2,14) = 1.931$, $p = 0.182$, $\eta_p^2 = 0.219$; Condition: $F(1,14) = 2.553$, $p = 0.132$, $\eta_p^2 = 0.154$; Timepoint*Condition: $F(2, 14) = 0.697$, $p = 0.515$, $\eta_p^2 = 0.094$). A significant effect of Condition on SICI was observed (Condition: $F(1,14) = 6.338$, $p = 0.024$, $\eta_p^2 = 0.309$), with SICI being more pronounced for 5Hz-rTUS (i.e., lower ratio) compared to sham. However, there was no significant interaction with Timepoint ($F(3,16) = 2.134$, $p = 0.136$, $\eta_p^2 = 0.288$), nor a main effect thereof ($F(3,17) = 0.971$, $p = 0.429$, $\eta_p^2 = 0.144$). No significant effects were observed for ICF (Timepoint: $F(3,14) = 0.916$, $p = 0.458$, $\eta_p^2 = 0.16$; Condition: $F(1,14) = 2.872$, $p = 0.112$, $\eta_p^2 = 0.17$ Timepoint*Condition: $F(3,16) = 0.856$, $p = 0.483$, $\eta_p^2 = 0.135$). Taken together, these results do not provide evidence for effective ultrasonic neuromodulation of corticospinal excitability.

RM-ANOVAs similarly did not reveal evidence for effective ultrasonic neuromodulation (*Raw MEP amplitude*: Timepoint: $F(3,39) = 3.133$, $p = 0.036$, $\eta_p^2 = 0.194$; Condition: $F(1,13) = 0.061$, $p = 0.808$, $\eta_p^2 = 0.005$; Timepoint*Condition: $F(3,39) = 1.445$, $p = 0.245$, $\eta_p^2 = 0.1$; *MEP amplitude as ratio to baseline*: Timepoint: $F(2,26) = 1.382$, $p = 0.269$, $\eta_p^2 = 0.096$; Condition: $F(1,13) = 1.489$, $p = 0.244$, $\eta_p^2 = 0.103$; Timepoint*Condition: $F(2,26) = 0.472$, $p = 0.629$, $\eta_p^2 = 0.035$; *SICI*: Timepoint: $F(3,42) = 3.185$, $p = 0.033$, $\eta_p^2 = 0.185$; Condition: $F(1,14) = 1.308$, $p = 0.272$, $\eta_p^2 = 0.085$; Timepoint*Condition: $F(1.66,23) = 0.439$, $p = 0.614$, $\eta_p^2 = 0.03$; *ICF*: Timepoint: $F(1.85,26) = 0.714$, $p = 0.489$, $\eta_p^2 = 0.049$; Condition: $F(1,14) = 0.49$, $p = 0.496$, $\eta_p^2 = 0.034$; Timepoint*Condition: $F(2.09,29) = 0.583$, $p = 0.572$, $\eta_p^2 = 0.04$).

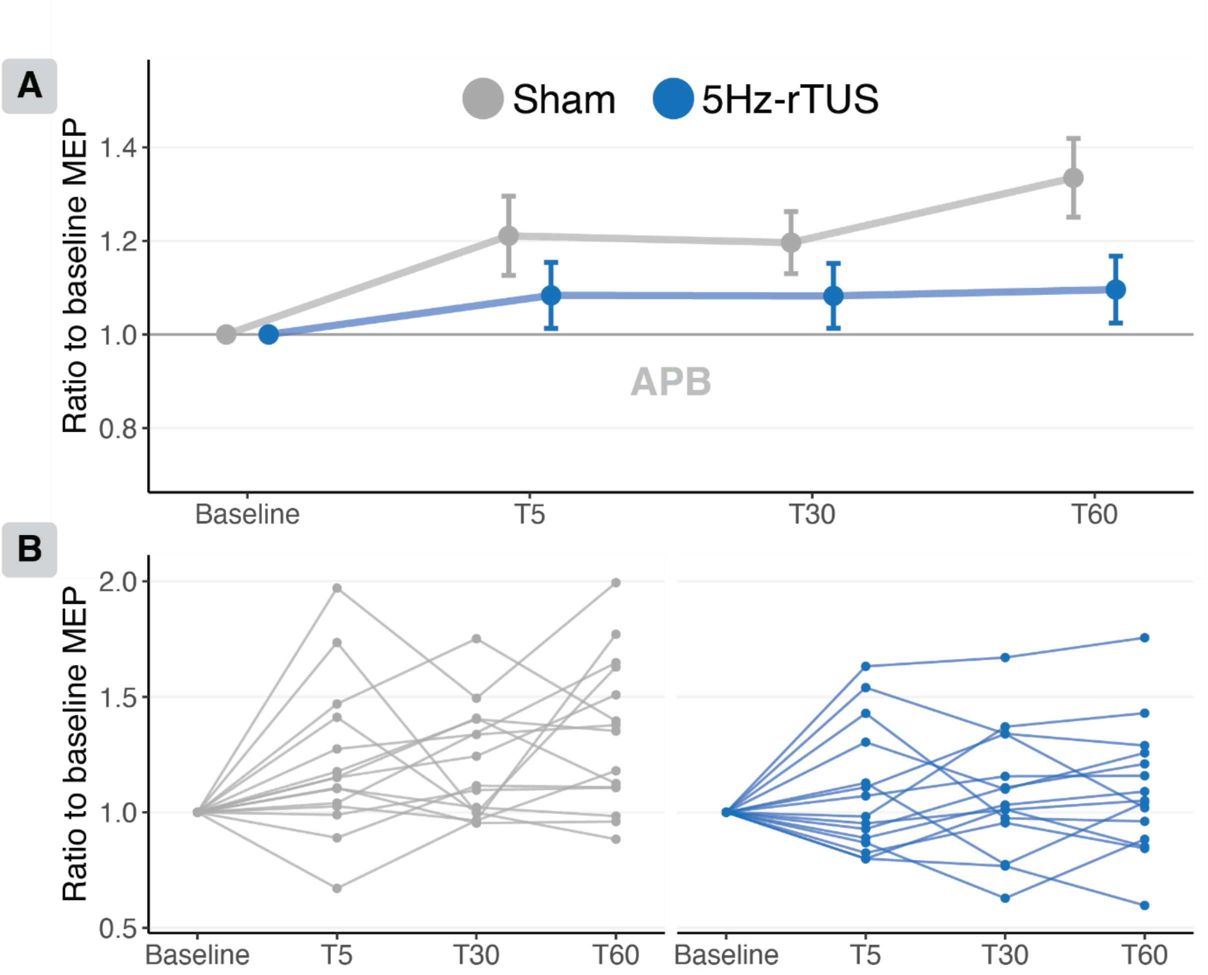

Supplementary Fig. 5. No significant effect of TUS on MEP amplitude measured over the adjacent abductor pollicis brevis (APB).

A. There was a significant main effect of Timepoint (Baseline/T0/T5/T30/T60), but no significant main effect or interaction with Condition (5Hz-rTUS/sham). MEP amplitudes are expressed as a ratio to baseline for each timepoint. Heavyweight points: Group mean ± standard error.

B. Participant-level data.

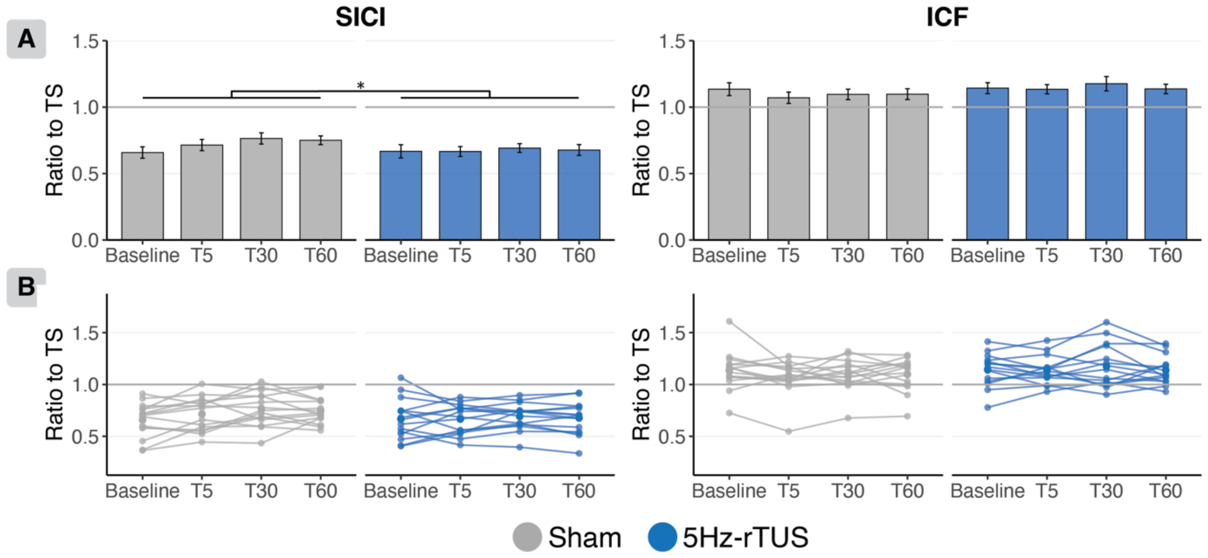

Supplementary Fig. 6. No significant excitatory effect of TUS on SICI or ICF for APB.

A. For SICI there was a significant effect of Condition (5Hz-rTUS/sham), but SICI was more pronounced for 5Hz-rTUS than sham, pointing towards inhibition rather than excitation. For ICF, there was no significant effect of TUS. MEP amplitudes are expressed as a ratio to the test-stimulus. Data and error bars represent group mean ± standard error.

B. Participant-level data.

Supplementary Information 4

Results for abductor digiti minimi (ADM)

A linear mixed model predicting square root corrected MEP amplitude measured over the ADM by Condition (5Hz-rTUS/sham), Timepoint (Baseline/T5/T30/T60) and their interaction revealed a significant effect of Timepoint ($F(3,14) = 4.674$, $p = 0.018$, $\eta_p^2 = 0.493$), with MEP amplitude increasing over time. No significant effect of Condition ($F(1,14) = 1.582$, $p = 0.229$, $\eta_p^2 = 0.101$) or Timepoint*Condition ($F(3, 14) = 1.396$, $p = 0.285$, $\eta_p^2 = 0.23$) was observed. When testing MEP amplitude expressed as a ratio to baseline, no significant effects were observed (Timepoint: $F(2,14) = 1.548$, $p = 0.247$, $\eta_p^2 = 0.179$; Condition: $F(1,14) = 0.874$, $p = 0.366$, $\eta_p^2 = 0.059$; Timepoint*Condition: $F(2, 14) = 2.118$, $p = 0.157$, $\eta_p^2 = 0.231$). Similarly, no significant effects were observed for either SICI or ICF (SICI: Timepoint: $F(3,16) = 2.219$, $p = 0.126$, $\eta_p^2 = 0.298$; Condition: $F(1,14) = 0.306$, $p = 0.589$, $\eta_p^2 = 0.022$; Timepoint*Condition: $F(3,16) = 0.04$, $p = 0.989$, $\eta_p^2 = 0.007$; ICF: Timepoint: $F(3,15) = 0.968$, $p = 0.434$, $\eta_p^2 = 0.165$; Condition: $F(1,14) = 1.875$, $p = 0.193$, $\eta_p^2 = 0.118$; Timepoint*Condition: $F(3,17) = 1.739$, $p = 0.196$, $\eta_p^2 = 0.231$).

RM-ANOVAs similarly did not reveal evidence for effective ultrasonic neuromodulation (*Raw MEP amplitude*: Timepoint: $F(1.45,19) = 2.948$, $p = 0.09$, $\eta_p^2 = 0.185$; Condition: $F(1,13) = 1.971$, $p = 0.184$, $\eta_p^2 = 0.132$; Timepoint*Condition: $F(1.58,21) = 1.834$, $p = 0.189$, $\eta_p^2 = 0.124$; *MEP amplitude as ratio to baseline*: Timepoint: $F(2,26) = 0.775$, $p = 0.471$, $\eta_p^2 = 0.056$; Condition: $F(1,13) = 0.352$, $p = 0.563$, $\eta_p^2 = 0.026$; Timepoint*Condition: $F(2,26) = 1.329$, $p = 0.282$, $\eta_p^2 = 0.093$; *SICI*: Timepoint: $F(1.68,23) = 0.539$, $p = 0.56$, $\eta_p^2 = 0.037$; Condition: $F(1,14) = 0.035$, $p = 0.854$, $\eta_p^2 = 0.003$; Timepoint*Condition: $F(1.84,26) = 0.657$, $p = 0.514$, $\eta_p^2 = 0.045$; *ICF*: Timepoint: $F(3,42) = 1.481$, $p = 0.234$, $\eta_p^2 = 0.096$; Condition: $F(1,14) = 0.368$, $p = 0.554$, $\eta_p^2 = 0.026$; Timepoint*Condition: $F(3,42) = 0.425$, $p = 0.736$, $\eta_p^2 = 0.029$).

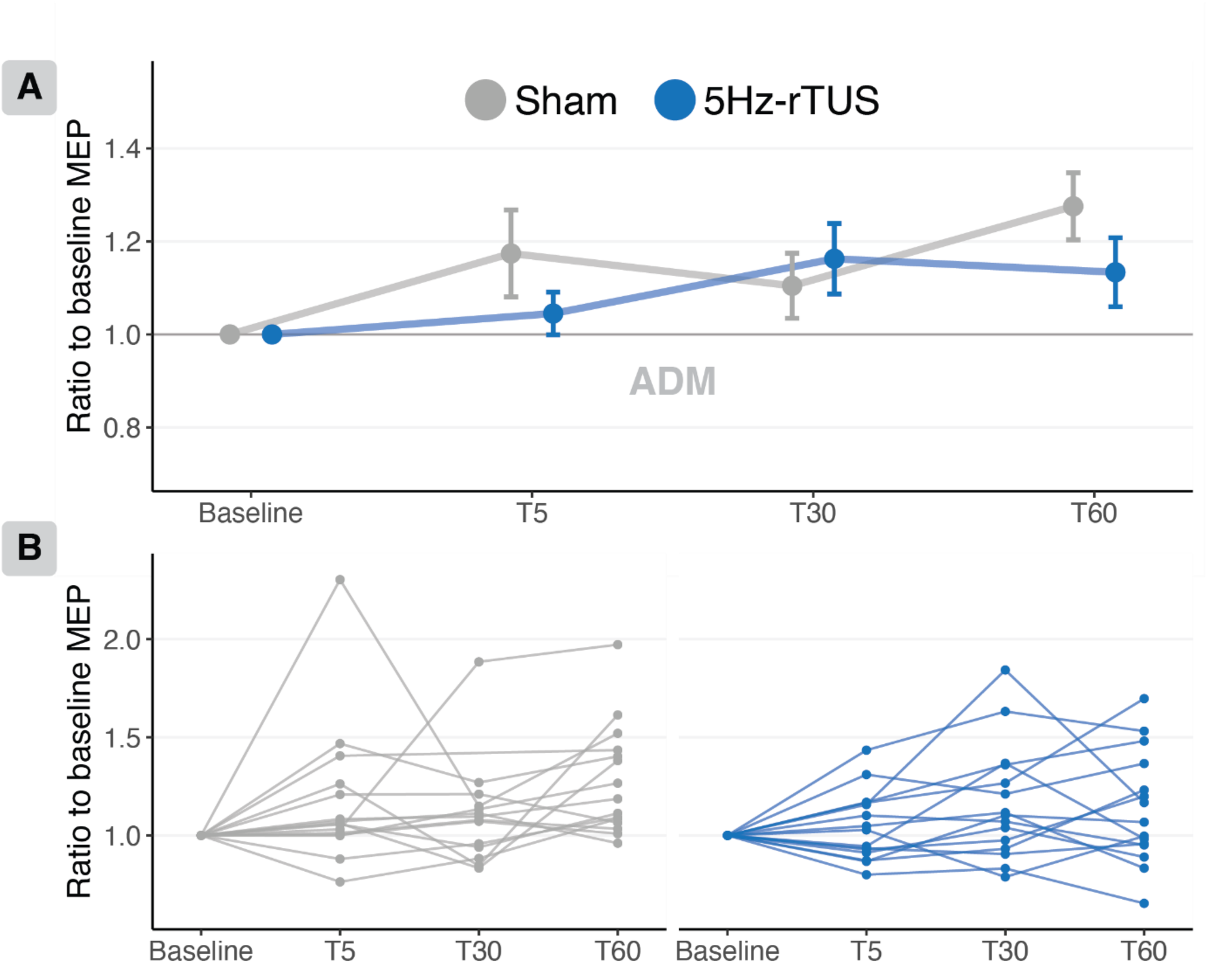

Supplementary Fig. 7. No significant effect of 5Hz-rTUS/sham on MEP amplitude measured over the adjacent abductor digiti minimi (ADM).

A. There was a significant main effect of Timepoint (Baseline/T0/T5/T30/T60), but no significant main effect or interaction with Condition (5Hz-rTUS/sham). MEP amplitudes expressed as a ratio to baseline for each timepoint. Points and error bars represent group mean ± standard error.

B. Participant-level data.

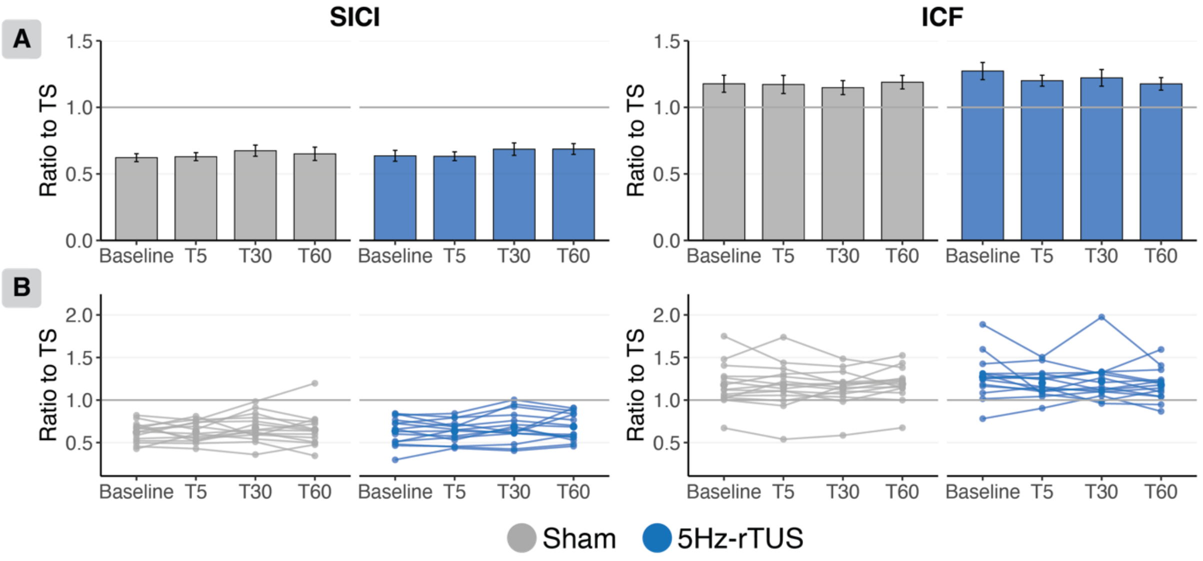

Supplementary Fig. 8. No significant effect of 5Hz-rTUS/sham on SICI or ICF. MEP amplitudes are expressed as a ratio to the test-stimulus.

A. For both SICI and ICF, there are no significant differences between sham and 5Hz-rTUS at any time point. Data and error bars represent group mean ± standard error.

B. Participant-level data.

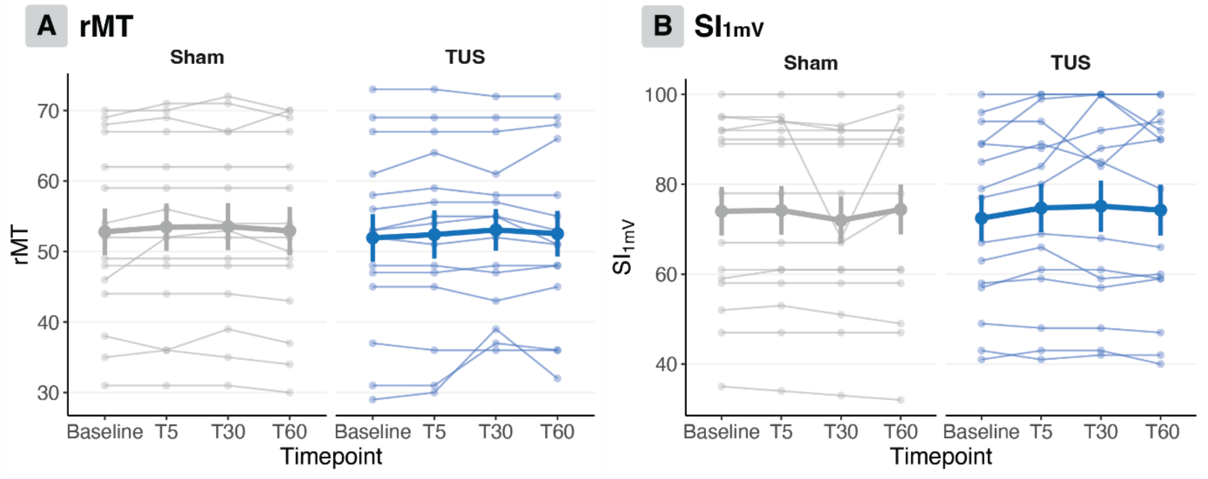

Supplementary Fig. 9. No significant effect of 5Hz-rTUS on resting motor threshold or SI_{1mV}

- **A.** No significant effect of 5Hz-rTUS on resting motor threshold (rMT). Data: Mean ± standard error. Participant-level data: smaller points and lines.
- **B.** No significant effect of 5Hz-rTUS on the stimulator intensity required to evoke a ~1mV MEP (SI_{1mV}). Heavyweight points and error bars represent group mean ± standard error. Lightweight points represent participant-level data.

Reference

Franbourg, A., Hallegot, P., Baltenneck, F., Toutain, C., & Leroy, F. (2003). Current research on ethnic hair. *J Am Acad Dermatol*, *48*(6 Suppl), S115-119. https://doi.org/10.1067/mjd.2003.277

Murphy, K. R., Nandi, T., Kop, B., Osada, T., Lueckel, M., N'Djin, W. A., Caulfield, K. A., Fomenko, A., Siebner, H. R., Ugawa, Y., Verhagen, L., Bestmann, S., Martin, E., Butts Pauly, K., Fouragnan, E., & Bergmann, T. O. (2025). A practical guide to transcranial ultrasonic stimulation from the IFCN-endorsed ITRUSST consortium. *Clin Neurophysiol*, *171*, 192-226. https://doi.org/10.1016/j.clinph.2025.01.004

Osada, T., Nakajima, K., Ogawa, A., Oka, S., Kamagata, K., Aoki, S., Oshima, Y., Tanaka, S., & Konishi, S. (2022). Distributions of cortical depth of the index finger region in the M1: A representative depth parameter for transcranial ultrasound stimulation. *Brain Stimul*, *15*(6), 1348-1350. https://doi.org/10.1016/j.brs.2022.09.012

Robbins, C. R. (2002). *Chemical and Physical Behavior of Human Hair*. Springer Verlag.

Yaakub, S. N., Bault, N., Lojkiewiez, M., Bellec, E., Roberts, J., Philip, N. S., Rushworth, M. F. S., & Fouragnan, E. F. (2024). Non-invasive Ultrasound Deep Neuromodulation of the Human Nucleus Accumbens Increases Win-Stay Behaviour. *bioRxiv*, 2024.2007.2025.605068. https://doi.org/10.1101/2024.07.25.605068

Yaakub, S. N., White, T. A., Roberts, J., Martin, E., Verhagen, L., Stagg, C. J., Hall, S., & Fouragnan, E. F. (2023). Transcranial focused ultrasound-mediated neurochemical and functional connectivity changes in deep cortical regions in humans. *Nat Commun*, *14*(1), 5318. https://doi.org/10.1038/s41467-023-40998-0

Zeng, K., Darmani, G., Fomenko, A., Xia, X., Tran, S., Nankoo, J. F., Shamli Oghli, Y., Wang, Y., Lozano, A. M., & Chen, R. (2022). Induction of Human Motor Cortex Plasticity by Theta Burst Transcranial Ultrasound Stimulation. *Ann Neurol*, *91*(2), 238-252. https://doi.org/10.1002/ana.26294
